# General Liquid‐Driven Coaxial Flow Focusing Preparation of Novel Microcapsules for Rechargeable Magnesium Batteries

**DOI:** 10.1002/advs.202002298

**Published:** 2020-11-27

**Authors:** Xirong Lin, Jinyun Liu, Haikuo Zhang, Yan Zhong, Mengfei Zhu, Ting Zhou, Xue Qiao, Huigang Zhang, Tianli Han, Jinjin Li

**Affiliations:** ^1^ National Key Laboratory of Science and Technology on Micro/Nano Fabrication Key Laboratory for Thin Film and Microfabrication of Ministry of Education Department of Micro/Nano‐electronics Shanghai Jiao Tong University Shanghai 200240 P. R. China; ^2^ Key Laboratory of Functional Molecular Solids of Ministry of Education Anhui Laboratory of Molecule‐Based Materials College of Chemistry and Materials Science Anhui Normal University Wuhu Anhui 241000 P. R. China; ^3^ National Laboratory of Solid State Microstructures Nanjing University Nanjing 210093 P. R. China

**Keywords:** energy storage, microcapsules, microfluidic method, secondary batteries

## Abstract

Magnesium batteries have been considered promising candidates for next‐generation energy storage systems owing to their high energy density, good safety without dendrite formation, and low cost of magnesium resources. However, high‐performance cathodes with stable capacity, good conductivity, and fast ions transport are needed, since many conventional cathodes possess a low performance and poor preparation controllability. Herein, a liquid‐driven coaxial flow focusing (LDCFF) approach for preparing a novel microcapsule system with controllable size, high loading, and stable magnesium‐storage performance is presented. Taking the MoS_2_‐infilled microcapsule as a case study, the magnesium battery cathode based on the microcapsules displays a capacity of 100 mAh g^−1^ after 100 cycles. High capacity retention is achieved at both low and high temperatures of −10, ‒5, and 45 °C, and a stable rate‐performance is also obtained. The influences of the liquid flow rates on the size and shell thickness of the microcapsules are investigated; and electron and ion diffusion properties are also studied by first‐principle calculations. The presented LDCFF method is quite general, and the high performance of the microcapsules enables them to find broad applications for making emerging energy‐storage materials and secondary battery systems.

## Graphical abstract

A liquid‐driven coaxial flow focusing micro‐fluidic method for preparing microcapsules which can be used in high‐performance secondary batteries is presented. The magnesium batteries based on the MoS_2_‐infilled microcapsules show stable capacities at different temperatures, and a recoverable rate performance.

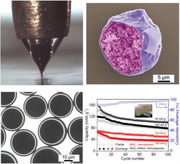

Currently, lithium‐ion batteries have dominated the global power source markets for portable electronics, electrical vehicles, etc.^[^ ^1^, ^2^, ^3^ ^]^ However, the limited lithium resources increase the cost; dendrite formation of metallic lithium leads to some safety risks; and their energy density needs to be higher than currently available.^[^ ^4^, ^5^, ^6^ ^]^ Those issues severely restrict the further development of lithium‐ion batteries,^[^ ^7^ ^]^ which inspires the investigations for some alternative energy storage systems, such as lithium‐sulfur,^[^ ^8^, ^9^ ^]^ sodium‐ion,^[^ ^10^, ^11^ ^]^ potassium‐ion,^[^ ^12^ ^]^ aluminum‐ion,^[^ ^13^ ^]^ and magnesium (Mg) batteries.^[^ ^14^ ^]^ Among them, rechargeable Mg batteries have attracted broad attention owing to their high theoretical capacity (3833 mAh cm^−3^ and 2205 mAh g^−1^), low electrode potential (‒2.36 V vs standard hydrogen electrode), abundance of Mg (10^4^ times more than lithium by weight), good chemical stability without dendrite formation, and environmental friendliness.^[^ ^15^, ^16^, ^17^, ^18^ ^]^ Mg batteries have been considered promising next‐generation energy storage systems. Nevertheless there are some challenges for Mg batteries currently:^[^ ^19^, ^20^ ^]^ (i) the kinetically slow intercalation and diffusion of Mg ions in cathodes result in a low capacity and poor rate‐performance; (ii) a stable high‐voltage electrolyte is needed. Seeking for high capacity, good rate capability and cycling stability cathode materials remain significant for the rechargeable magnesium batteries. Recently, several efforts have been contributed to address those issues. For example, Sun et al. prepared a sodium vanadium bronze Na_2_V_6_O_16_·1.63H_2_O cathode via one‐step hydrothermal process.^[^ ^21^ ^]^ After cycling 450 times at a current density of 200 mA g^−1^, a reversible capacity of 65 mAh g^−1^ was obtained. It was considered the structural water acted as a pillar, providing good structural stability and fast ion mobility. In addition, an environmentally friendly, low‐cost, and sustainable triazine‐based porous covalent organic framework cathode for Mg storage was also reported.^[^ ^22^ ^]^ It delivered a high power density of 2.8 kW kg^−1^ and a high specific energy density of 146 Wh kg^−1^, which was ascribed to the porous framework structure.

Among many improving approaches for the Mg battery cathodes, such as nanostructing as mentioned above, encapsulating storage materials inside a conductive and confined environment would be able to enhance the performance by improving the conductivity without affecting the intrinsic properties of the active materials. However, conventional preparation approaches, such as emulsification,^[^ ^23^, ^24^ ^]^ templated synthesis,^[^ ^25^ ^]^ and layer‐to‐layer self‐assembly,^[^ ^26^ ^]^ are time‐exhaust. The equipment and operation steps are complicated, and reactions are sensitive to temperature and pH value, leading to poor controllability and repeatability, and low yield. Compared to them, liquid‐driven coaxial flow focusing (LDCFF) technology^[^ ^27^, ^28^ ^]^ is able to prepare monodisperse microdroplets with uniform size distribution, designated loading, high encapsulation efficiency and productivity, and good controllability. Recently, LDCFF approach has been used in pesticide biomedicine, diagnosis, and drug delivery. Zhong et al. fabricated a pesticide‐loaded microcapsule via the LDCFF process.^[^ ^29^ ^]^ The release of pesticides has sustained release profiles, which depends on the core–shell structure and shell degradation. For instance, Zhu et al. encapsulated concentrated indocyanine green (ICG) in monodisperse bilayer liposomes for quantitative fluorescence imaging and drug delivery.^[^ ^30^ ^]^ So far, there is rare report about using LDCFF to prepare electrode materials for secondary batteries.

Herein, we present a novel and general LDCFF approach to fabricate microcapsules for Mg storage. As a case study, typical two‐dimensional (2D) layered structure molybdenum disulfide (MoS_2_) was encapsulated inside a microcapsule system for Mg battery cathodes to improve the conductivity and electrochemical performance. The experimental setup of the LDCFF method contains three syringe pumps, a stainless steel coaxial needle, a pressure chamber, a glass plate with small holes and a monitor, as illustrated in Figures S1 and S2 (Supporting Information). The flow rates of the inner phase (*Q*_i_) which is the MoS_2_ nanospheres solution, the outer phase (*Q*_o_) photocurable organic phases, and the focusing phase (*Q*_f_) are controlled by three syringe pumps. As *Q*_f_ reaches the threshold, the inner and outer fluids contact in a coaxial cone between the needle and orifice. The coaxial liquid jet eventually breaks up into droplets because of the flow instability.^[^ ^31^ ^]^ The collected droplets containing MoS_2_ nanospheres and shell precursor were solidified by UV light radiation, then the samples were calcined after freezer‐drying, finally forming the microcapsules with MoS_2_ nanospheres as core and a carbon shell. In our study, the Mg battery cathodes based on the MoS_2_‐infilled microcapsules show a high and stable capacity of 100 mAh g^−1^ after cycling 100 times, along with a Coulombic efficiency exceeding 99%. The microcapsules also exhibit a good capacity at temperatures of ‒10, ‒5, and 45 °C, indicating a potential for practical applications. In addition, a possible reaction mechanism for the reversible process of Mg^2+^ ion insertion/extraction has been investigated using first‐principle calculations.

**Figure** **1**a shows the optical images of the LDCFF process for fabricating MoS_2_‐infilled microcapsules. Under specific multiphase flow rates, the high‐speed focusing fluid elongates the outer and inner fluids into a coaxial cone near the orifice. Because of the flow instability, the focused coaxial jet breaks into uniform microdroplets at the orifice exit, along with a MoS_2_ suspension encapsulated by the outer liquid (ethoxylated trimethylolpropane triacrylate, ETPTA). The breakup of the jet is caused by the propagation of disturbances on the interface.^[^ ^32^ ^]^ The real‐time preparation videos are presented in Movies S1 and S2 (Supporting Information). Movies S3–S5 (Supporting Information) show the detailed information of Movie S2 (Supporting Information) with a high‐speed photographic industrial camera. Figure 1b,c displays the scanning electron microscope (SEM) and transmission electron microscopy (TEM) images of the MoS_2_ nanospheres prepared through a hydrothermal route, respectively, which are used as the core of capsules. The MoS_2_ exhibits a three‐dimensional (3D) nanospherical morphology with a diameter of ≈400 nm. Each nanosphere is assembled by several nanoflakes. The interplanar distance of the MoS_2_ nanoflake is 0.62 nm (Figure S3a, Supporting Information), corresponding to the *d*‐spacing of (002) planes.^[^ ^33^ ^]^ The well‐defined rings in the selected area electron diffraction (SAED) pattern (Figure S3b, Supporting Information) are indexed to the (100) and (110) planes of the hexagonal MoS_2_ (JCPDS card no. 37‐1492).^[^ ^34^, ^35^ ^]^

**Figure 1:**
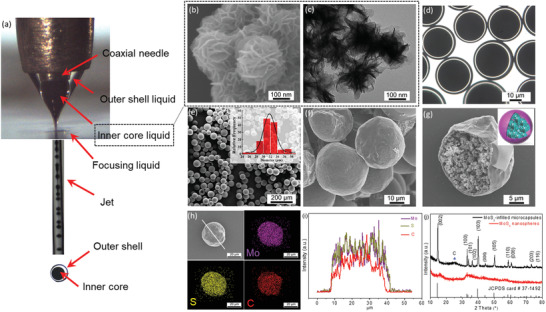
a) A steady cone‐jet mode in the LDCFF process. b) SEM and c) TEM image of the MoS_2_ nanospheres. The d) optical and e) SEM image of the MoS_2_‐infilled microcapsules after UV‐solidification. The inset in (e) shows the size‐distribution. f,g) SEM images of the MoS_2_‐infilled microcapsules. h) SEM image and elemental mappings of Mo, S, C. i) The distribution of elements from the line‐scanning in (h). j) XRD patterns.

In Figure 1d and Figure S4a (Supporting Information), since the MoS_2_ suspension is opaque, the collected microdroplets are black, as observed on an optical microscopy. The SEM images (Figure S4b, Supporting Information and Figure 1e) show the microcapsules are smooth after solidification by UV radiation. Under the conditions of *Q*_i_ = 3 mL h^−1^, *Q*_o_ = 4 mL h^−1^ and *Q*_f_ = 700 mL h^−1^, the average diameter of the microcapsules is about 30 µm, as displayed in Figure 1e and the inset. Figure 1f confirms the stable spherical morphology of the capsules after thermal carbonization. Seen from a broken microcapsule (Figure 1g and Figure S4c, Supporting Information), MoS_2_ nanospheres encapsulated inside the capsule are observed, whereas the shell thickness of the capsule is ≈300 nm. The porous structure of the microcapsule shell is confirmed by the pore‐size distribution (Figure S4d, Supporting Information), which is beneficial for the electrolyte penetration. In order to study the distribution of elements within the MoS_2_‐infilled microcapsules, the SEM and elemental mapping images of a microcapsule were recorded, as shown in Figure 1h. Elemental mapping indicates a uniform dispersion of the elements S, Mo, and C. In addition, the energy‐dispersive X‐ray (EDX) spectrum is shown in Figure S5 (Supporting Information). In the line‐scanning profiles (Figure 1i), the peaks of Mo, S, and C are presented. The peak width of each element is ≈32 µm, which is in good agreement with the capsule size shown in Figure 1e. X‐ray diffractometer (XRD) patterns are presented in Figure 1j. Compared to the pure MoS_2_ nanospheres, the diffraction peaks of the MoS_2_‐infilled microcapsules become narrow and sharp, indicating an enhanced crystallization of the MoS_2_ after heat treatment.^[^ ^36^ ^]^

Thermal gravimetric analysis (TGA) was conducted from room temperature to 600 °C in air to determine the carbon content of the MoS_2_‐infilled microcapsules, as shown in Figure S6 (Supporting Information). For comparison, the weight loss for the pure MoS_2_ nanospheres is about 29.1%, which is attributed to the oxidation of MoS_2_ into MoO_3_.^[^ ^37^, ^38^ ^]^ For the MoS_2_‐infilled microcapsules, the weight remains about 47.5% after thermal treatment, which is from the MoO_3_. Since that, the MoS_2_ within the microcapsules is ≈53 wt%. The loading is controllable by adjusting the preparation conditions, which will be presented below.

To investigate the chemical composition and state of the MoS_2_‐infilled microcapsules, X‐ray photoelectron spectroscopy (XPS) analysis was performed. The survey spectrum indicates that the main elements include Mo, S, and C (Figure S7a, Supporting Information), which are consistent with the EDX results. The Mo 3d spectra show two peaks at 229.7 and 232.8 eV, which are assigned to Mo 3d_5/2_ and Mo 3d_3/2_, respectively, indicating the existence of Mo^4+^.^[^ ^39^ ^]^ In addition, a S 2s peak is located at 226.9 eV (Figure S7b, Supporting Information).^[^ ^40^ ^]^ The peaks at 162.6 and 163.7 eV correspond to the S 2p_3/2_ and S 2p_1/2_ of S^2−^ in MoS_2_ (Figure S7c, Supporting Information), respectively.^[^ ^33^ ^]^ In Figure S7d (Supporting Information), the high‐resolution of C 1s can be deconvoluted into two peaks. The peak located at 284.6 eV is ascribed to the C‒C, while the one at 285.3 eV is attributed to the sp^3^ hybridized carbon.^[^ ^41^ ^]^

The overall size and shell thickness of the microcapsules can be controlled easily by varying the flow rates. In previous studies,^[^ ^30^, ^42^ ^]^ the LDCFF process follows a scaling law, as presented below: *D* ∼ *α* [(*Q*_i_ + *Q*_o_)/*Q*_f_]^1/2^·*D*_orif_, where *α* stands for a constant dependent on the process parameters such as the liquid properties; *D* and *D*_orif_ represent the diameters of the droplet and orifice, respectively; *Q*_i_, *Q*_o_, and *Q*_f_ are the flow rates of the inner, outer, and focusing fluids, respectively.

The diameter of the microcapsules increases depending on the increase of the *Q*_i_, *Q*_o_, and *Q*_i_ + *Q*_o_; while it decreases with the increase of *Q*_f_, as shown in Figures S8–10 (Supporting Information) and **Figure** **2**i–ii. For example, in Figure S8b (Supporting Information), *Q*_i_ = 3 mL h^−1^, *Q*_o_ = 4 mL h^−1^, when the rates of *Q*_f_ is 300 mL h^−1^, the diameter of capsules is 168 µm; however, when *Q*_f_ increase to 1000 mL h^−1^, the diameter of capsules decreases to 49 µm. In Figure 2i and Figure S9 (Supporting Information), the liquid flow rates of *Q*_f_ is constant at 700 mL h^−1^ and *O*_i_ = *Q*_o_. When *O*_i_ = *Q*_o_ = 2 mL h^−1^ increased to 5, 8, 9, 11, 14, 17, and 20 mL h^−1^, the capsule size gradually increased, the Taylor cone is elongated by the roundness and fullness to be closer to the orifice. In Figure 2ii and Figure S10 (Supporting Information), the liquid flow rates of *Q*_i_ is 3 mL h^−1^ and *Q*_o_ is 4 mL h^−1^. When *O*_f_ = 200 mL h^−1^ increased to 300, 400, 600, 800, 1000, and 1200 mL h^−1^, the capsule size decreased step by step, and Taylor cone becomes tenuous. Good agreement is achieved between the experimental and theoretical results for a given *α* = 1.886.

**Figure 2:**
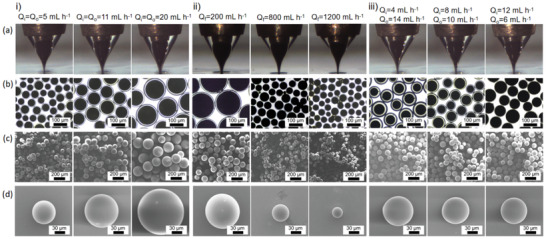
a) Sequence of experimental images showing the cone‐jet structure changes. b) The microscopic, c) low‐ and d) high‐magnification SEM images of the produced microdroplets changes. For (i), the liquid flow rates: *O*_i_ = *Q*_o_, *Q*_f_ = 700 mL h^−1^. For (ii), the liquid flow rates: *O*_i_ = 3 mL h^−1^, *Q*_o_ = 4 mL h^−1^. For (iii), the liquid flow rates: *Q*_i_ + *Q*_o_ = 18 mL h^−1^, *Q*_f_ = 700 mL h^−1^.

The stability of the cone‐jet configuration under different flow rate ratios between internal and external phases (*ϕ* = *Q*_i_/*Q*_o_) is shown in Figure 2iii and Figure S11 (Supporting Information). The thickness is associated with the diameters of microcapsules and the proportion of *Q*_i_/*Q*_o_. The scaling law shows when the microdroplets has one core, it is expressed as:^[^ ^29^ ^]^ *d*∼[1−*Q*_i_^1/3^/(*Q*_i_ + *Q*_o_)^1/3^]·*D*, where *d* is the average shell thickness, *D* is the diameters of microcapsules, *Q*_i_ and *Q*_o_ are the flow rates of inner and outer liquid, respectively. The shell thicknesses of the microcapsules can be adjusted by changing the flow rate ratio. Figure 2a(iii) and Figure S11a (Supporting Information) show the optical images of the cone‐jet morphology changing with *ϕ* (2:16, 4:14, 6:12, 8:10, 10:8, 12:6, 14:4) when *Q*_i_ + Q_o_ = 18 mL h^−1^, *Q*_f_ = 700 mL h^−1^. As the flow rate ratio decreases, the inner Taylor cone becomes thin while the shell thickness turns to be thick (Figure S12, Supporting Information). When the flow rate ratio exceeds a certain threshold which is 0.286 in our study, the prepared microdroplets have one core only, as shown in Figure 2b with the liquid flow rates: *Q*_i_ = 4 mL h^−1^, *Q*_o_ = 14 mL h^−1^, *Q*_f_ = 700 mL h^−1^. However, when the flow rate ratio is less than 0.125, the microdroplets have multiple cores, as shown in Figure S11b (Supporting Information).

The Mg‐storage properties of the MoS_2_‐infilled microcapsules are shown in **Figure** **3**. Figure 3a displays the 1st, 2nd, 3rd, 50th, and 100th galvanostatic charge–discharge curves of the microcapsules‐based cathode at a current density of 50 mA g^−1^. A plateau at 1.3 V in discharge is attributed to the Mg^2+^ intercalation. The plateau at ≈1.7 V in charge is assigned to Mg^2+^ deintercalation.^[^ ^43^ ^]^ During the first cycle, the discharge and charge capacities are 161 and 144 mAh g^−1^, respectively. The cycling performance of the MoS_2_‐infilled microcapsules and nanospheres is shown in Figure 3b. At the current densities of 50 and 100 mA g^−1^, the MoS_2_‐infilled microcapsules provide a discharge capacity of 161 mAh g^−1^ in the first cycle, which remains 100 mAh g^−1^ after 100 cycles. The Coulombic efficiency keeps exceeding 99%, as shown in Figure S13 (Supporting Information). As seen, the capacities of the MoS_2_‐infilled capsules are obviously higher than the pure MoS_2_ nanospheres, exhibiting an enhanced Mg‐storage performance by the encapsulation. Furthermore, the capacities of the capsules presented here also exceed the capsules with a lower MoS_2_ loading of 34.8%, as displayed in Figure S14 (Supporting Information). In Table S1 (Supporting Information), the MoS_2_‐infilled microcapsules show an improved performance compared to some other reports. The inset in Figure 3b shows the fabricated battery is able to light up the light‐emitting diodes (LEDs) after charge–discharge 100 times.

**Figure 3:**
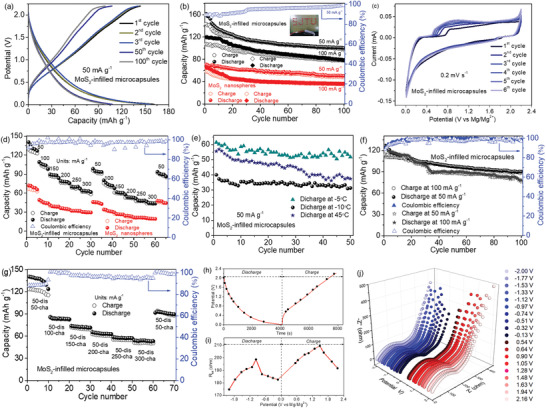
a) Charge–discharge profiles of the MoS_2_‐infilled microcapsules‐based cathode at 50 mA g^−1^. b) Cycling capacity and Coulombic efficiency of the microcapsules‐based Mg battery inset show a photo of LEDs powered by the microcapsules‐based battery after cycling 100 times. c) CV curves of the microcapsules at a sweep rate of 0.2 mV s^−1^. d) Rate performance. e) Cycling capacities of the microcapsule cathode at 45, ‒5, and ‒10 °C, respectively. f) Cycling performance of the MoS_2_‐infilled microcapsules charging at 100 mA g^−1^ and discharging at 50 mA g^−1^, charging at 50 mA g^−1^, and discharging at 100 mA g^−1^. g) Capacity and Coulombic efficiency as the charge rate was changed and the discharge rate was kept at 50 mA g^−1^. h) Selected potentials for measuring EIS spectra. i) Relationship of *R*_tot_ versus potential. j) Nyquist plots measured at different potentials within a cycle.

Figure 3c displays the cyclic voltammetry (CV) profile of the MoS_2_‐infilled microcapsules‐based cathode. The MoS_2_ undergoes reversible Mg^2+^ intercalation and deintercalation, corresponding to the cathodic peak at 1.25 V and the anodic peak at 1.75 V, respectively. In contrast, the MoS_2_ nanospheres without being encapsulated show a weak current without apparent redox in Figure S15 (Supporting Information). The reaction mechanism is described as follows:^[^ ^44^ ^]^

(1) $$\begin{array}{c} \mathrm{Cathode}:\;6\mathrm{Mo}S_{2}+4Mg^{2+}+8e^{-}↔Mg_{4}Mo_{6}S_{12} \end{array}$$

(2) $$\begin{array}{c} \mathrm{Anode}:\;4\mathrm{Mg}↔4Mg^{2+}+8e^{-} \end{array}$$

(3) $$\begin{array}{c} \mathrm{Overall}:\;6\mathrm{Mo}S_{2}+4\mathrm{Mg}↔Mg_{4}Mo_{6}S_{12} \end{array}$$

The rate‐performances of the MoS_2_‐infilled microcapsules and pure MoS_2_ nanospheres at 50, 100, 150, 200, 250, and 300 mA h^−1^ are displayed in Figure 3d. The measurements were repeated three times. For example, in the second round, when the current density was turned back to 50 mA h^−1^, the capacity of the MoS_2_‐infilled microcapsules recovered to 94 mAh g^−1^. In contrast, the pure MoS_2_ nanospheres only showed a capacity of 48 mAh g^−1^. In order to evaluate the potential for practical applications, the MoS_2_‐infilled microcapsules‐based batteries were measured at temperatures of 45, ‒5, and ‒10 °C under 50 mA g^−1^, respectively. In Figure 3e, when cycling at a relatively high temperature of 45 °C, the first‐cycle capacity is about 56 mAh g^−1^, then remains 37 mAh g^−1^ after 50 cycles. The MoS_2_‐infilled microcapsules exhibit a capacity of 52 mAh g^−1^ after cycling 50 times at ‒5 °C. At ‒10 °C, the capacity remains 31 mAh g^−1^ at the 50th cycle, along with a capacity retention of 75.5%. Similar results, to the best of our knowledge, are rarely reported in literature for rechargeable magnesium‐ion batteries. The cycling at different charge versus discharge rates were performed, as shown in Figure 3f. After cycling 100 times, capacities for the two cases of charge/discharge rates of 100/50 mA g^−1^ and 50/100 mA g^−1^ remain 90 and 78 mAh g^−1^, respectively. In addition, a series of cycles at different charge rates under the same discharge rate (Figure 3g) were measured. When the discharge rate was maintained at 50 mA g^−1^, the capacity exhibited a decreasing trend with an increasing rate. Once the cycling charge and discharge rates were turned back to 50 mA g^−1^, the capacity recovered to a high level with a capacity retention rate of 74.1%, indicating a potential for using at different conditions.

To better understand the enhancement mechanism of the MoS_2_‐infilled microcapsules compared to the pure MoS_2_ nanospheres, electrochemical impedance spectroscopy (EIS) measurements were conducted. Figure S16 (Supporting Information) shows the EIS spectra and the equivalent circuit model. The fitting results indicate that the charge transfer resistances (*R*_ct_) values, which correspond to the diameters of the semicircles in the spectra,^[^ ^45^ ^]^ for the microcapsules and the pure MoS_2_ nanospheres are 205.6 and 516.7 Ω, respectively. It indicates that the microcapsule structure is able to obviously improve the conductivity, which is beneficial for a high electrochemical performance.^[^ ^46^ ^]^ EIS measurements at different potentials were carried out to study the influence of the Mg ion diffusion during the charge and discharge through the microcapsules. Figure 3h shows the initial Mg ion insertion/extraction processes. The total resistance (*R*_tot_) which is the sum of the interface and charge transfer resistances is presented in Figure 3i.^[^ ^47^ ^]^ The spectra at different charge and discharge potentials were continuously measured, as shown in Figure 3j. It has a relatively high value at initial stage, then the *R*_tot_ decreases as the discharge potential decreases. At the charge process, the resistance increases significantly depending on the increase of the potential. The total resistance of the last stage is close to the same as the initial one, indicating a good reversibility.

The electrochemical kinetics were investigated by a series of CV measurements at various scanning rates of 0.1–1.0 mV s^−1^, as shown in **Figure** **4**a. Depending on the increase of scanning rate, the peak current increases. The peak currents (*i*) and rates (*v*) follow the equations:^[^ ^48^ ^]^ *i* = *av* ^b^, and log(*i*) = *b*log(*v*) + log(*a*). The coefficient *b* reflects the charge‐storage kinetics of the electrode. The *b* = 1.0 implies the electrode is controlled by capacitive response, while *b* = 0.5 indicates the diffusion process is dominated.^[^ ^49^ ^]^ In Figure 4b, the calculated *b* for the cathodic and anodic processes are 0.81 and 0.93, respectively, indicating a substantial capacity of capacitance contribution. The proportions of the capacitive contribution at different rates are presented in Figure 4c. The ratios increase depending on the increase of rates. A large portion of capacitive contribution implies that the microcapsule structure provides rapid kinetics for Mg insertion/extraction.^[^ ^50^ ^]^

**Figure 4:**
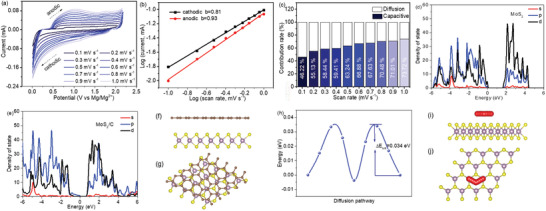
a) CV profiles at different rates. b) The log(*i*) versus log(*v*) plots. c) Ratios of the capacitive contributions at different rates. Density of states of d) pure MoS_2_ and e) MoS_2_ with carbon. f) The side‐ and g) top‐view of MoS_2_ with carbon layer. h) Mg ion diffusion energy on MoS_2_. i) The side‐ and j) top‐view of Mg ion diffusion pathway.

In addition, DFT calculations were employed to study the transfer of electrons and ions. In Figure 4d, the MoS_2_ possesses a band gap of around 1.7057 eV, indicating a semi‐conductivity property. However, when it is interacted with carbon (Figure 4e), the band gap decreases to 0.6843 eV, which indicates an increased conductivity. This result proved that wrapping MoS_2_ with carbon can effectively facilitate electron transport. The result proves that wrapping MoS_2_ with carbon effectively facilitates the electron transport. Mg ions transfer on the MoS_2_ was also investigated. In the side and top views in Figure 4i,j, Mg ions are prone to situate at the top of Mo atom. When they transfer to the next top site, Mg ions go through a hexagon center. In this process, the energy difference of the two sites forms the diffusion barrier energy, which is 0.034 eV for the Mg diffusion barrier energy on MoS_2_, as shown in Figure 4h. The small diffusion barrier is able to accelerate the fast Mg ions transport, enabling a good electrochemical performance. In addition, the SEM images of the microcapsules after cycling 100 times were obtained, as shown in Figure S17 (Supporting Information). The capsule structure keeps well, and the nanoflowers are encapsulated in the capsules, indicating a robust structure of the microcapsule system during charge‐discharge.

In summary, we present a novel LDCFF approach for preparing a microcapsule system with high performance of Mg storage. As a case study, the size of the prepared MoS_2_‐infilled microcapsules can be easily controlled by adjusting the flow rates of inner, middle, and outer phases. The MoS_2_‐infilled microcapsules exhibit a good electrochemical stability and a high electron transfer kinetics. The capsules‐based Mg battery cathode shows a stable capacity of 100 mAh g^−1^ with a high Coulombic efficiency exceeding 99% after 100 cycles at 50 mA g^−1^. The rate‐performance of the MoS_2_‐infilled microcapsules keeps stable after repeated three times, which is much better than the pure MoS_2_ without being encapsulated. In addition, at low temperatures of ‒5 and ‒10 °C, and relatively high temperature of 45 °C, the microcapsules also provide stable capacities. The good performance is ascribed to the carbon shell, which is able to provide a protection for the core materials from loss, and enhances the conductivity that has been demonstrated by DFT calculations on DOSs. It is expected that the the microcapsule design presented here with controllable and general preparation approach, and the good electrochemical performance will be of great significance for preparing many other emerging energy‐storage materials and high‐performance secondary battery systems. The presented LDCFF method also provides a potential for preparing microcapsules with different sizes, structures, and material combinations for various applications such as in medicine and pharmacy, encapsulating drugs into microcapsules that meet specific clinical needs. In future more studies are required, such as the preparation of small‐size microcapsules and the mechanism of interface chemistries for different core and shell of capsule systems.

## Conflict of Interest

The authors declare no conflict of interest.

## Author Contributions

J.Y.L. proposed the conceptual approach. X.L., J.Y.L., and J.J.L. designed the experiments. X.L., H.Z., T.H., Y.Z., M.Z., T.Z., X.Q., and H.Z. carried out the experiments and analysis. X.L., J.Y.L., and J.J.L. wrote the manuscript and revised it with contributions from all coauthors. J.Y.L. and J.J.L. supervised the investigation.

## Supporting information

Supporting Information

Supplemental Video 1

Supplemental Video 2

Supplemental Video 3

Supplemental Video 4

Supplemental Video 5
