# Supplementary material for: General Liquid‐Driven Coaxial Flow Focusing Preparation of Novel Microcapsules for Rechargeable Magnesium Batteries
Source: Adv Sci (Weinh). 2020 Nov 27;8(2):2002298. doi: 10.1002/advs.202002298 (PMC7816708; doi:10.1002/advs.202002298)
Supplement: Supplementary file 1 — Supporting Information [file ADVS-8-2002298-s001.pdf]

© 2020 Wiley-VCH GmbH

ADVANCED SCIENCE Open Access

Supporting Information

for *Adv. Sci.,* DOI: 10.1002/advs.202002298

General liquid-driven coaxial flow focusing preparation of novel microcapsules for rechargeable magnesium batteries

Xirong Lin, Jinyun Liu, Haikuo Zhang, Tianli Han, Yan Zhong, Mengfei Zhu, Ting Zhou, Xue Qiao, Huigang Zhang, and Jinjin Li**

Supporting Information

General liquid-driven coaxial flow focusing preparation of novel microcapsules for rechargeable magnesium batteries

Xirong Lin, Jinyun Liu, Haikuo Zhang, Tianli Han, Yan Zhong, Mengfei Zhu, Ting Zhou, Xue Qiao, Huigang Zhang, and Jinjin Li**

Experimental Section

Synthesis of MoS_2 nanospheres. The MoS_2 nanospheres were synthesized with a hydrothermal method. 0.865 g of ammonium molybdate and 0.87 g of thioacetamide were dissolved in 30 ml distilled water under stirring. 0.5 g of methoxypolyethylene glycol was added into the solution and the mixture was continue stirred for 1 h. The resulting solution was transferred into a 50 ml Teflon-lined stainless steel autoclave and reacted for 3 h at 180 ºC. After cooling to room temperature, the black precipitates were washed with deionized water for three times combined with centrifugation.

LDCFF process for Generation of MoS_2-infilled microcapsules. The coaxial needle consists of an inner needle (inner diameter: 0.26 mm, outer diameter: 0.49 mm) and an outer needle (inner diameter: 0.84 mm, outer diameter: 1.27 mm) joined together in parallel by the laser welding method. Meanwhile, the tip of the inner needle is 0.01-0.2 mm longer than that of the outer needle. The coaxial needle assembly is held by a rubber plug and inserted into the pressure chamber (inner diameter: 19 mm, outer diameter: 25 mm, length: 18 mm). The outlet of the coaxial needle faces a small orifice (diameter: 0.3 mm) located at the center of a thin glass plate and the vertical distance is adjusted to be 1.2 mm. Figure S2 illustrates these details clearly.

The inner aqueous phase consists of MoS_2 nanospheres ultrasound dispersed in (vinyl alcohol) (PVA). Furthermore, the focusing phase is 2 wt% PVA solution. The outer phase is

photocurable organic phases employing ethoxylated trimethylolpropane triacrylate (ETPTA) resin with 5% photoinitiator 2-hydroxy-2-methylpropiophenone. Firstly, 1 g of photoinitiator and 20 g of ethyl alcohol were mixied homogeneously. Then the mixed solution was added into to 20 g of ETPTA drop by drop under stirring. After 0.5 h, the mixture was placed in an oven for 12 h at 70 ºC to promote complete volatilization of the ethyl ethanol.

The flow rates of the Q_i=3 mL h^{-1}, Q_o=4 mL h^{-1} and Q_f= 700 mL h^{-1} were controlled by syringe pumps (Suzhou Xunfei Scientific Instrument Co. LTD, XF-101P). The process is continuously monitored by a CCD camera (The Imaging Source, DFK-23G274) equipped with a microscopic lens. The illumination is provided by a strobe flashlight (Hangzhou Pintuo Electronic Technology Co. LTD, flashing frequency: 3.0 kHz) from the other side of the chamber. The small droplets were received which are MoS_2 microcapsules, and the collector is used to receive the samples. In order to prevent the microcapsules from cracking, a small amount of 10 wt% PVA solution was first charged into the collector. Meanwhile, the sample in the collector was solidified by UV light for 5 min illumination. Then the MoS_2 microcapsules are washed several times with distilled water and then dried overnight in a vacuum freeze dryer (LGJ-10). The freeze-dried samples were annealed at 500 °C for 2 h in purity N_2 atmosphere at a ramp rate of 2 °C min^{-1}.

Characterization. The morphology and structure were characterized using SEM (FEI QUANTA 200FEG), TEM (Hitachi HT7700), Nikon inverted microscope (ECLIPSE Ts2), XRD (Philips X'Pert) with a high intensity Cu Kα radiation (1.54178 Å). Elemental mappings were performed on the FIB (AURIGA) equipped with an Oxford INCA energy-dispersive X-ray (EDX) analyzer. The component of fabricated samples was analyzed using XPS (Thermo-VG Scientific ESCALAB 250), TGA (Perkin-Elmer, Pyris 1) at a ramp rate of 10 °C min^{-1} in air. Pore-size distribution was measured on a Tristar II 3020M system.

Electrochemical measurements. Electrochemical tests were carried out using CR2032 coin

cells assembled in an Ar-filled glove box (Mikrouna Super 1220/750, O_2 and H_2O < 0.01 ppm). The cathode were fabricated by mixing active materials (80 wt%), acetylene black (10 wt%) and poly-vinylidene fluoride (PVDF, 10 wt%) binder in N-methyl-pyrrolidinone (NMP) solvent to form a homogeneous slurry, and then the mixtures were coated on a 304 stainless steel foil, dried in a vacuum oven at 60 °C for 12 h. A glass fiber filter was employed as the separator. The 0.4 M $(PhMgCl)_2$-$AlCl_3$/THF (APC) electrolyte was synthesized to be used as the electrolyte. AZ31 Mg alloy was used as the counter and reference electrode, which was cut into wafers and polished with sandpaper to remove oxides and impurities on the surface. The electrolyte and Mg alloy were all prepared at in above glove box. The charge/discharge cycling were performed on a Neware battery tester (Shenzhen Neware Technology Co., Ltd, CT-3008). Cyclic voltammetry (CV) and Electrochemical impedance spectroscopy (EIS) were performed on a CHI-660E electrochemical workstation. CV curves were record over the potential range of 0.01-2.2 V (vs. Mg/Mg^{2+}). The EIS data was obtained by applying an AC voltage of 5 mV in the frequency range between 100 kHz and 500 mHz. For the tests at high and low temperatures (45 ºC, –5 ºC, and –10 ºC), a temperature-control system equipped with a chamber (DSP-HT, Shenzhen Disi Equipment Co., Ltd.) was used, which were set at the temperatures of 45 ºC, –5 ºC, and –10 ºC, respectively. The tester clips holding the coin cells in the chamber were connected with the Neware battery tester through extended lines. Before each test, the coin cells were put in the chamber for 24 h. After that, tests were started up. The coin cells were in the chamber at constant temperature during the charge-discharge process.

Simulation details. To study the electronic properties and Mg ion diffusion barrier energy of the sample, density functional theory calculations were conducted by using a Vienna Ab-initio Simulation Package (VASP). The ion-electron interaction was described by the projected augmented wave method, and Perdew-Burke-Ernzerhof (PBE) method was used in the generalized gradient approximation (GGA). The cutoff energy was set as 500 eV and a k-point mesh of 3×3×1 was used. In the calculations, the structures were optimized until the

force and energy less than 0.01 eV/Å and 1.0×10^{-6} eV/atom. In order to avoid the interaction between layers, a vacuum of 15 Å was added. Moreover, the climbing image nudged elastic band method was performed to analyze the Mg ion diffusion.

Statistical analysis. The size distribution of the microcapsules was measured by image-processing program (ImagePro Plus version 6.0 software, Media Cybernetics, Inc., USA). All results were expressed as mean ± S.D. Data were analysed on an OriginPro software (Origin Lab, Northampton, MA, USA). The coefficient of variation (C.V, C.V=S.D./Mean×100%) is used to represent the dispersion degree of data.

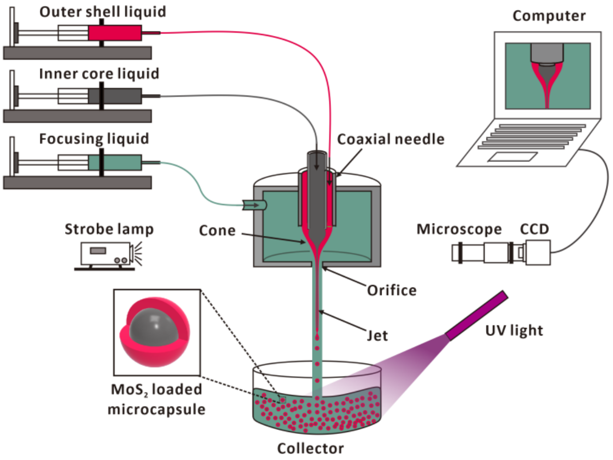

Figure S1. Schematic illustration of the LDCFF process and the production of the MoS_2-infilled microcapsules.

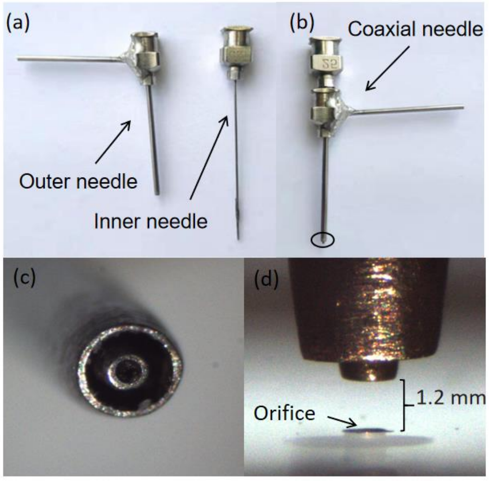

Figure S2. (a) The picture of inner and outer stainless steel needle. (b) The picture of coaxial needle. (c) Coaxial needle head magnification. (d) The distance between a small orifice on a thin plate and a coaxial needle.

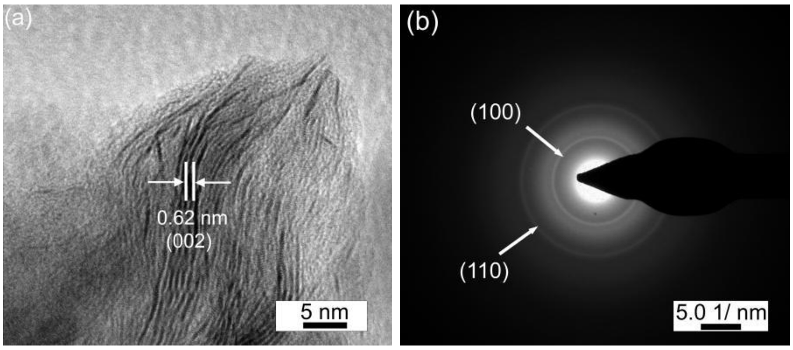

Figure S3. (a) HRTEM image and (b) corresponding SAED pattern of the MoS_2 nanospheres.

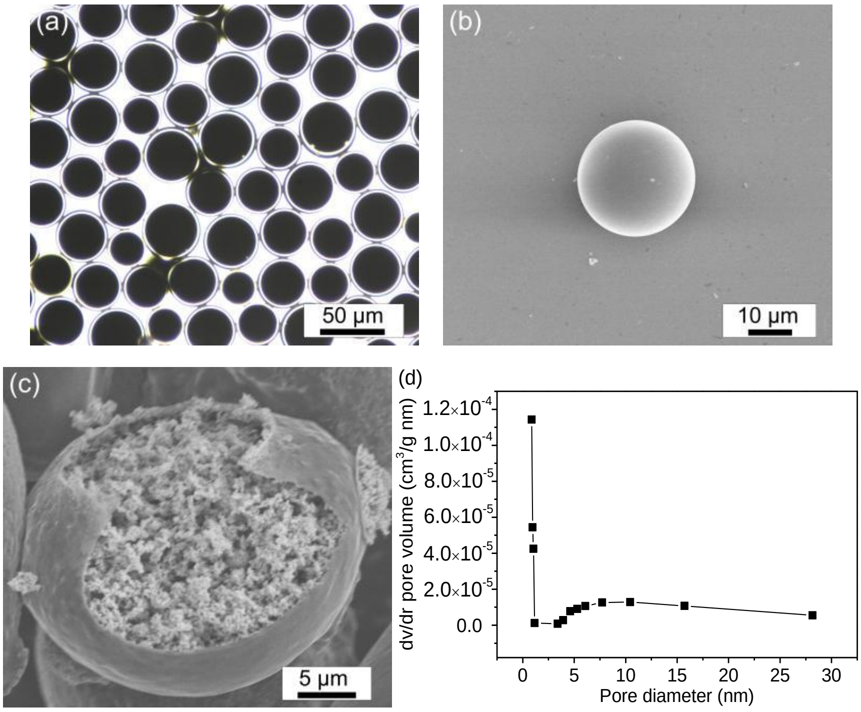

Figure S4. The (a) optical and (b) SEM image of the MoS_2-infilled microcapsules after solidification. (c) SEM image and (d) pore-size distribution of the microcapsules.

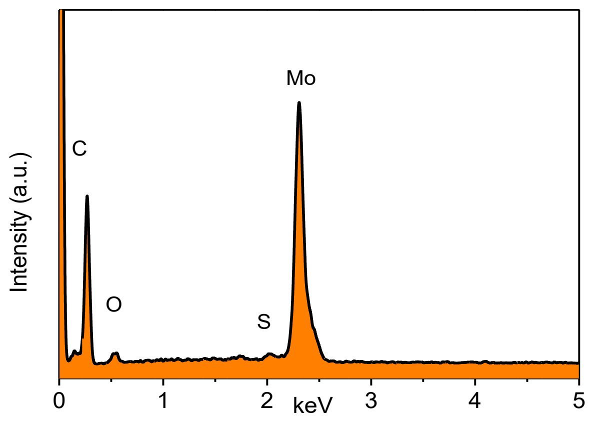

Figure S5. The EDX spectrum of the MoS_2-infilled microcapsules.

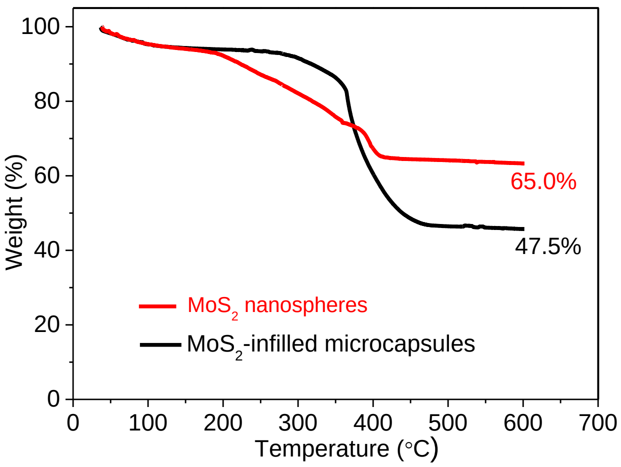

Figure S6. TGA profiles of the MoS_2-infilled microcapsules and nanospheres.

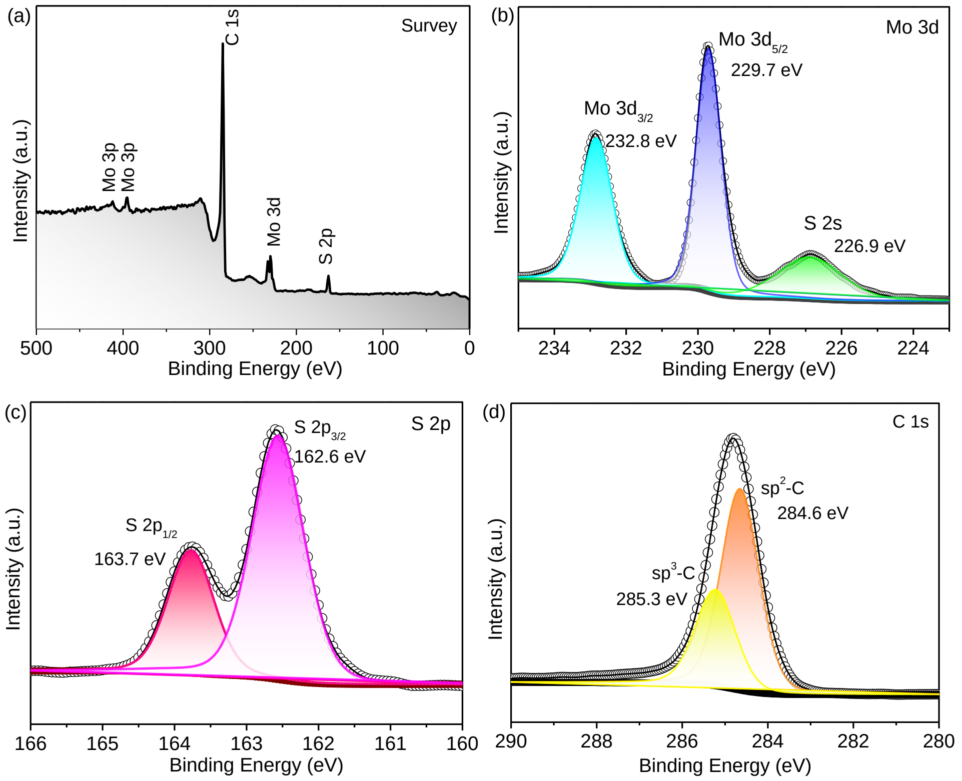

Figure S7. XPS spectra of the MoS_2-infilled microcapsules: (a) survey spectrum, (b) Mo 3d, (c) S 2p, and (d) C 1s.

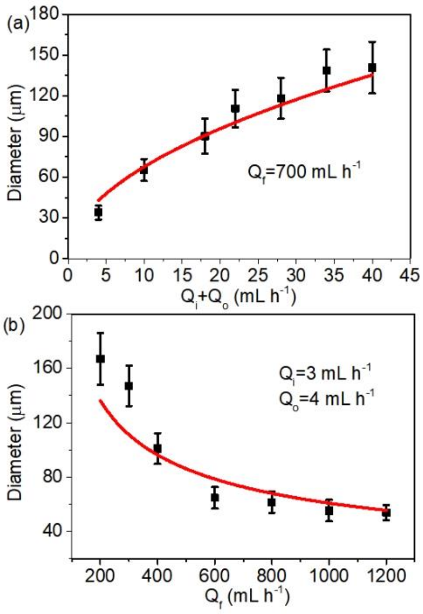

Information of MoS_2-infilled microcapsules diameter changes with $Q_i + Q_o$ for Q_f =700 mL h^{-1}

Q_i+Q_o (mL/h)	4	10	18	22	28	34	40
Sample size (n)	200	200	200	200	200	200	200
Mean	33.8	65.3	90.1	110.5	118.1	138.6	140.6
± S.D.	4.12	8.08	12.85	13.75	15.05	15.34	19.05
C. V (%)	12.2	12.4	14.3	12.4	12.7	11.1	13.5

Information of MoS_2-infilled microcapsules diameter changes with Q_f for Q_i =3 mL h^{-1}, Q_o = 4 mL h^{-1}

Q_f (mL/h)	200	300	400	600	8000	1000	1200
Sample size (n)	200	200	200	200	200	200	200
Mean	116.9	147.2	101.0	64.8	61.6	55.37	53.9
± S.D.	19.03	14.87	11.30	7.92	7.77	7.91	5.59
C. V (%)	11.4	10.1	11.2	12.2	12.6	14.3	10.4

Figure S8. Size control of the MoS_2-infilled microcapsules by changing Q_i, Q_o, and Q_f. (a) Droplet diameter D changes with Q_i+Q_o for Q_f=700 mL h^{-1} and Q_i=Q_o; (b) droplet diameter D changes with Q_f for Q_i=3 mL h^{-1}, Q_o=4 mL h^{-1}. The table at right side of each curve shows the corresponding information about the diameter variations.

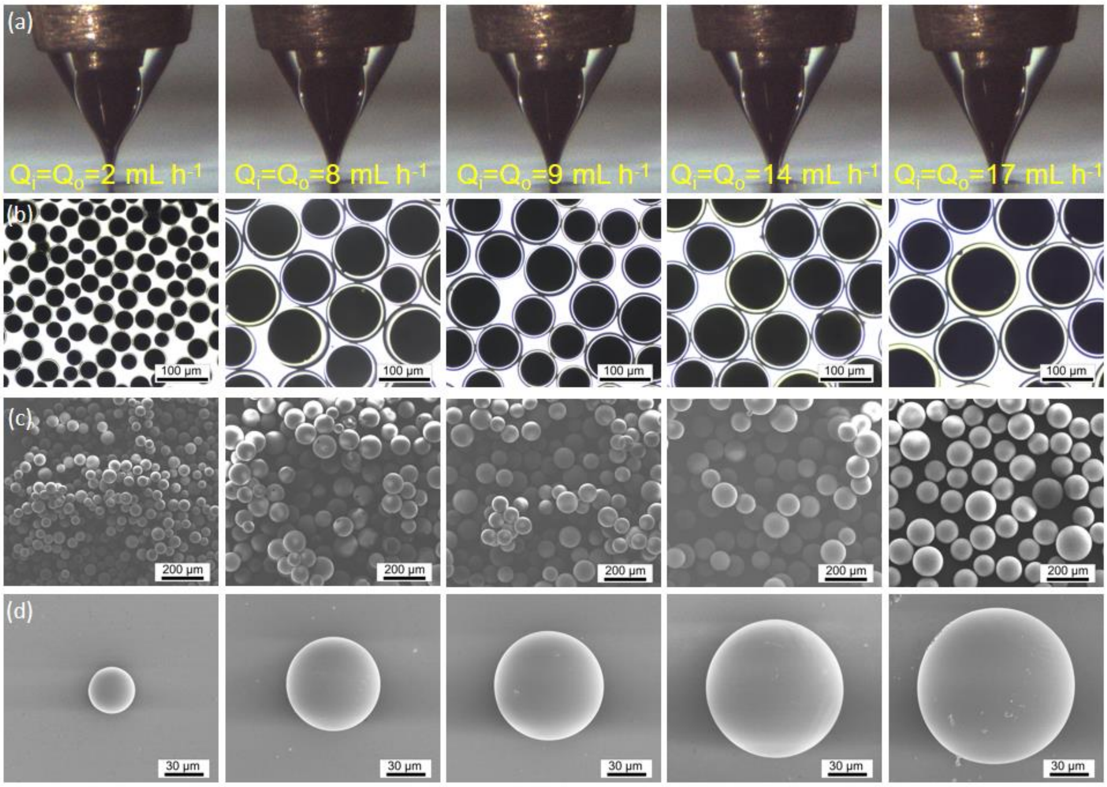

Figure S9. (a) Sequence of experimental images showing the cone-jet structure changes. (b) The microscopic, (c) low- and (d) high-magnification SEM images of the produced microdroplets changes. The liquid flow rates: O_i=Q_o, Q_f=700 mL h^{-1}.

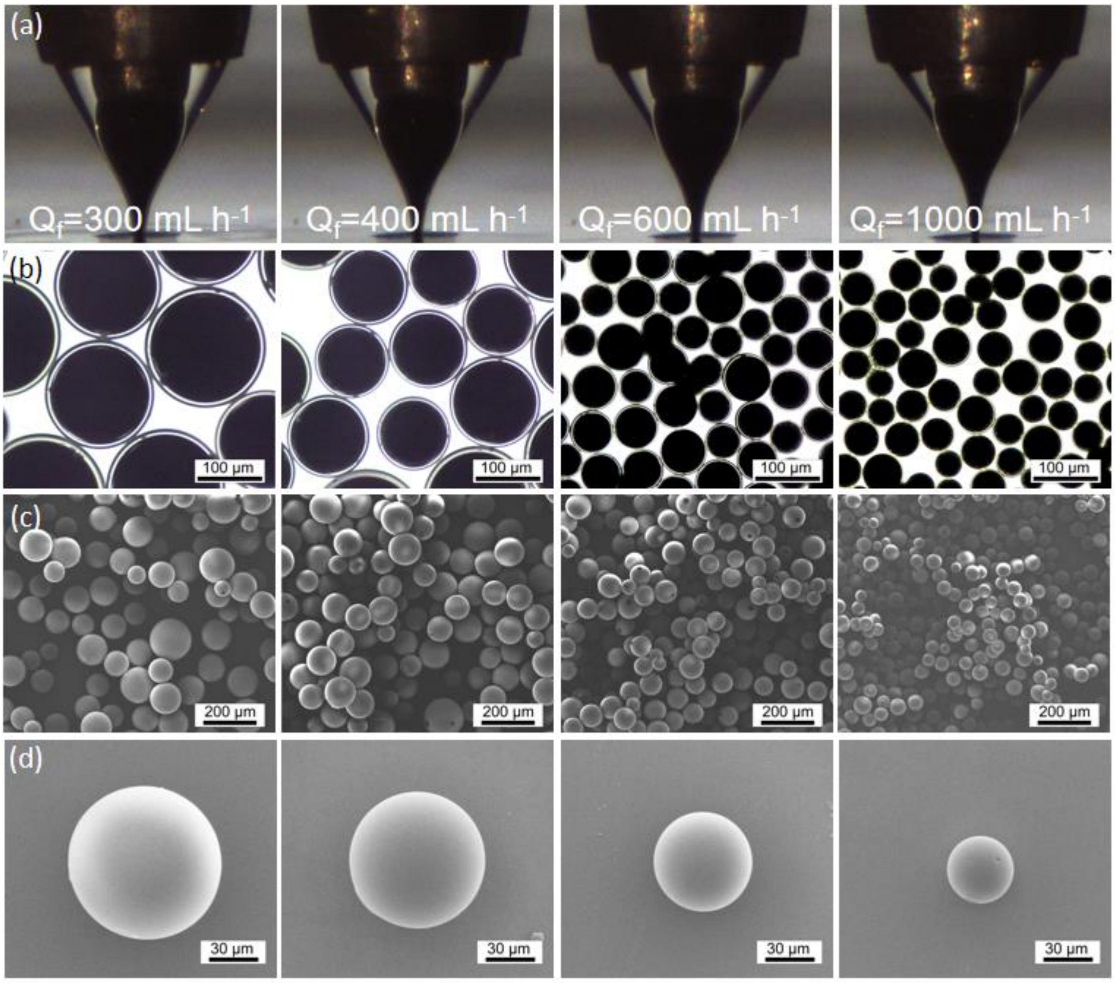

Figure S10. (a) Sequence of experimental images showing the cone-jet structure changes. (b) The microscopic, (c) low- and (d) high-magnification SEM images of the produced microdroplets changes. The liquid flow rates: O_i=3 mL h^{-1}, Q_o=4 mL h^{-1}.

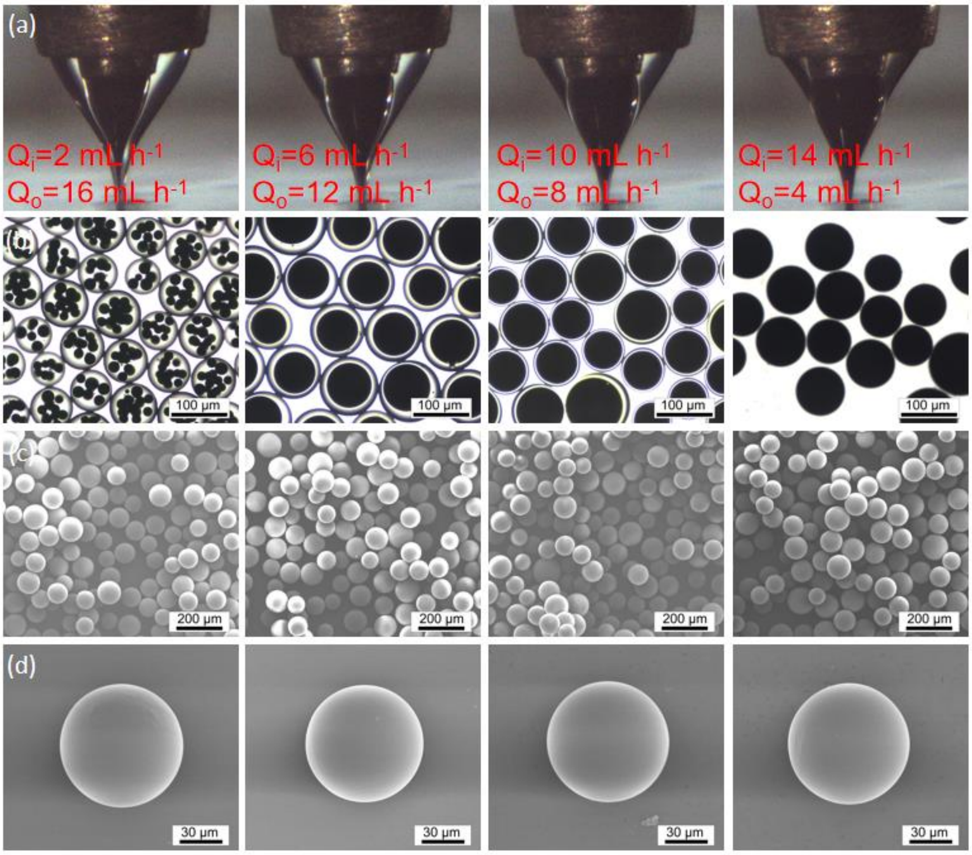

Figure S11. Shell thickness control of MoS_2-infilled microcapsules by changing flow rate ratios of inter and outer phases ($\varphi=Q_i/Q_o$). (a) Sequence of experimental images showing the cone-jet structure changing with φ. (b) The microscopic, (c) low- and (d) high- magnification SEM images of the produced microdroplets changing with φ. The liquid flow rates: Q_i+Q_o=18 mL h^{-1}, Q_f=700 mL h^{-1}.

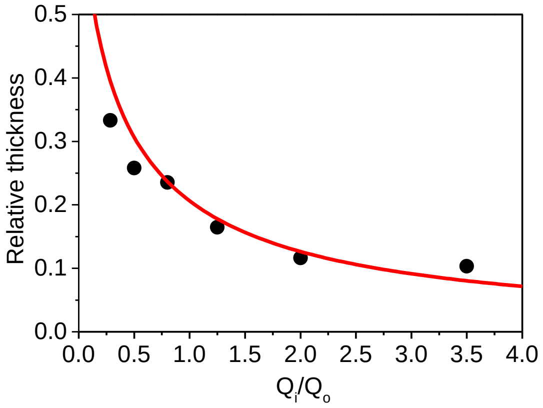

Figure S12. Relationship of relative thickness vs. Q_i/Q_o.

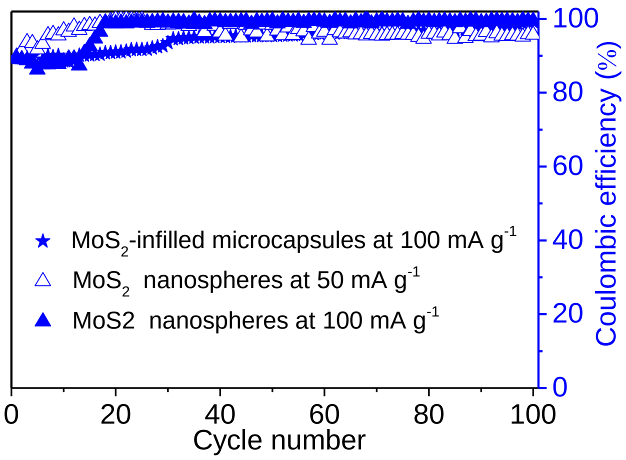

Figure S13. Coulombic efficiency of the samples.

Table S1. Comparison on the electrochemical performance of MoS_2-based cathodes.

Materials	Preparation method	Current density (mA g^{-1})	Cycle number	Capacity (mAh g^{-1})	Refs.
MoS_2/C microspheres	hydrothermal	50	50	84	1
MoS_2/rGO microspheres	hydrothermal	20	50	74	2
F-doped MoS_2	chemical solution	15	50	39	3
MoS_2/graphene	lithium-assisted sonication	20	50	82	4
MoS_2/graphene	hydrothermal	20	100	88	5
PEO-pillared MoS_2	atomic-level lattice engineering	5	30	70	6
MoS_2/MXene	hydrothermal	50	50	108	7
MoS_2-infilled microcapsules	LDCFF	50	100	100	This work

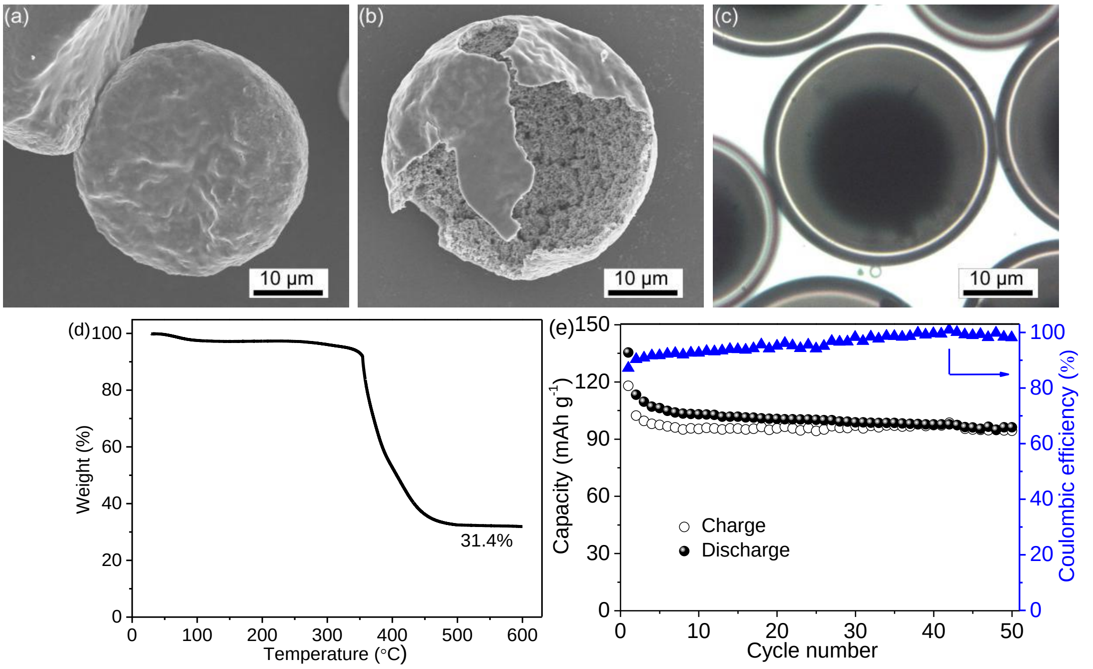

Figure S14. (a), (b) SEM and (c) optical image of MoS_2-infilled microcapsules with the liquid flow rates: Q_i=3 mL h^{-1}, Q_o=4 mL h^{-1}, Q_f=700 mL h^{-1}. (d) TGA curve. (e) Cycling performance.

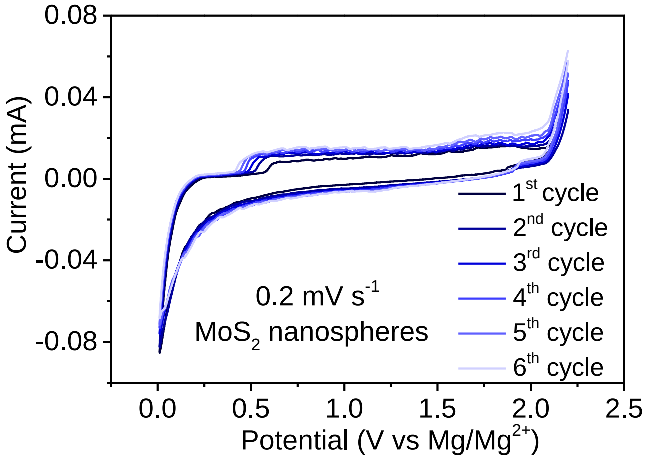

Figure S15. CV curves of MoS_2 nanospheres at a scan rate of 0.2 mV s^{-1}.

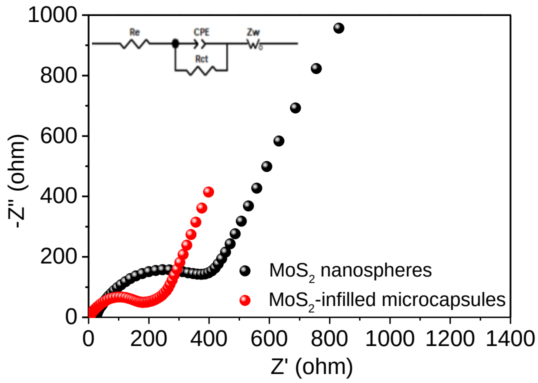

Figure S16. Nyquist plots of MoS_2-infilled microcapsules and nanospheres in fresh cells. The inset shows the equivalent circuit.

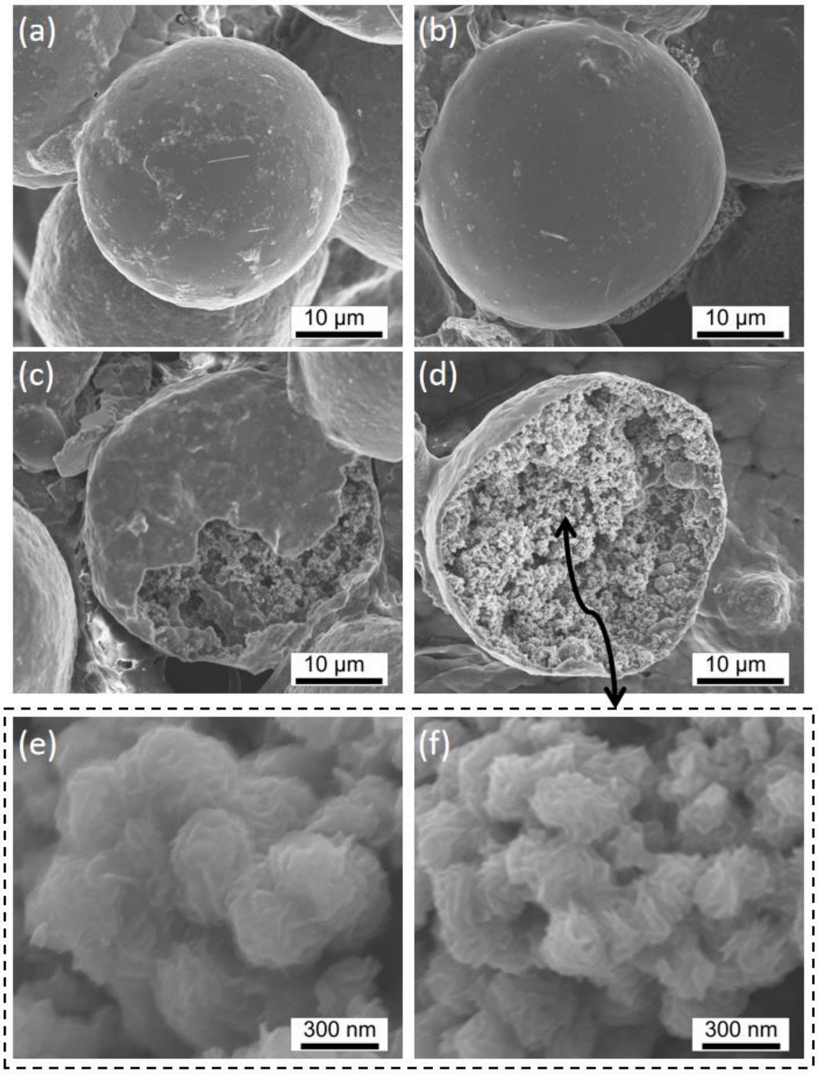

Figure S17. (a-f) SEM images of the MoS_2-infilled microcapsules after 100 cycles at 50 mA g^{-1}.

References

[1] Y. C. Liu, L. F. Jiao, Q. Wu, J. Du, Y. P. Zhao, Y. C. Si, Y. J. Wang, H. T. Yuan, *J. Mater. Chem. A* **2013**, *1*, 5822.

[2] Y. C. Liu, L. F. Jiao, Q. Wu, Y. P. Zhao, K. Z. Cao, H. Q. Liu, Y. J. Wang, H. T. *Nanoscale* **2013**, *5*, 9562.

[3] G. Venkateswarlu, D. Madhu, J. V. Rani, *Funct. Mater. Lett*. **2019**, *12*, 1950041.

[4] Y. C. Liu, L. Z. Fan, L. F. Jiao, *J. Power Sources* **2017**, *340*, 104.

[5] X. Fan, R. R. Gaddam, N. A. Kumar, X. S. Zhao, *Adv. Energy Mater*. **2017**, *7*, 1700317.

[6] Y. L. Liang, H. D. Yoo, Y. F. Li, J.. Shuai, H. A. Calderon, F. C. R. Hernandez, L. C. Grabow, Y. Yao, *Nano Lett.* **2015**, *15*, 2194.

[7] M. Xu, N. Bai, H. X. Li, C. Hu, J. Qi, X. B. Yan, *Chinese Chem. Lett.* **2018**, *29*, 1313.
